# The Hospitals

**Published:** 1874-07

**Authors:** 


### The Hospitals.

Cook County Hospital. May 16. Dr. H. M. Lyman. *Empyema-Paracentesis Thoracis.* Reported for Chicago Medical Journal by Ralph E. Starkweather, M.D.

The patient, a teamster thirty-five years of age, had been neglected before admission to the hospital. The breathing was costal, with movement of chest walls greatly impaired, and considerable emaciation. The line of resonance anteriorly, on right side, was above the third rib; posteriorly, on the right side, it was on a line parallel with the inferior border of the scapula. The right chest measured twenty inches; the left, nineteen inches. The respirations were forty, and pulse one hundred and twenty the minute; but, after tapping, respirations fell to twenty and pulse to one hundred the minute. The patient was tapped by Dr. Steele twice, on the right side, by means of the aspirator. The punctures were made at the angle of the rib, in the seventh intercostal space; and evacuated, the first time, ninety-six ounces, and a week later, eighty ounces, of thick and bloody pus. Usually the sixth intercostal space is selected by Dr. Steele as the seat of puncture.

In a case of pleurisy of the right side, and pneumonia of the left side, in the second week Dr. Lyman was giving the patient five grains of the muriate of ammonia every four hours, in syrup glycyrrhiza, besides quinine and Dover's powder, and continuous application of the jacket poultice.

Rush Medical College. May 21. Dr. Freer. *The Operation of Transfusion.*

In the presence of a number of the delegates to the State Medical Society, Dr. Freer explained the above operation. He first exhibited Dr. Aveling's method—the immediate, from vein to vein. It consisted in taking blood from a healthful person, and passing it, undefibrinated, by means of an india-rubber ball syringe, having the valves removed, and tubes of a foot in length, to an exsanguinated patient. The Doctor criticised the method unfavorably, saying that after four or five charges of blood the tubes attached to the bulb, and the canules also, would clog up; that there was danger from clots, and that it was objectionable to have to deal, while injecting, with a second person; and that trouble, haste and confusion would be inevitable. Dr. Freer preferred the plan proposed by his friend, Dr. McDonald, of Dublin, which he skillfully and brilliantly demonstrated on the body of a dog of about fifty pounds weight.

The apparatus of Dr. McDonald is more simple than that of Dr. Aveling, and consists of a large glass pipette, a piece of rubber tubing, a blunt-pointed, hollow needle or canule, opening a little distance from its blunt point. The dog having been etherized, had so much blood taken from him that most of the audience considered him as actually dead. The blood was defibrinated by rapidly whipping it with twigs taken from a brush-broom, and poured into the large pipette; thence a piece of rubber tubing conducted the blood to the canule, which was held fast in the opened vein. The blood, by its own weight and atmospheric pressure, flowed into the body, and speedily the dog revived and was able to walk about the room.

The median cephalic vein in the human body is to be chosen, the incision in the skin to be parallel with the vein; a tongue-shaped slit in the vein should be made from below upwards; and the tube should be held firmly to the vein, that no air may enter. Plenty of time should be taken for the injection. The blood must be defibrinated, and of a certain temperature. In case of hæmorrhage from placenta prævia, or post partum hæmorrhage, or accidental hæmorrhage, transfusion will prove to be invaluable.

*Epithelioma of the Left Foot.*

The patient, a young colored girl, exhibited extensive destruction, of two years progress, of the outer half of the dorsum of the foot; the tensor tendons were involved, together with the two smaller toes; there was also an abscess and fistulous tract in the middle plantar region. Dr. Bogue stated that he would remove a portion of the side of the foot, the little toe and its neighbor, including the distal half of the two metatarsal bones, and open the fistulous tract and abscess. The operation was done, the patient taking ether.

*Perinephritic Abscess.*

The patient, a man of thirty years, had been sick the past month; had lately had a chill followed by fever, loss of appetite and strength; a swelling in the left lumbar region, tender and painful.

Upon explorative puncture, there was a thick dense fluid which ran very slowly from the needle, though it was of a large size, and upon the fluid afterwards cooling, it would not run at all.

Dr. Bogue made an unusually deep incision, parallel with and at the external edge of the quadratus lumborum, and upon pushing aside the muscles, which were much infiltrated, pus gushed up. An india-rubber tube, perforated in numerous places near the end, one-eighth inch in diameter, was then inserted to the bottom of the abscess, that there might be free exit and drainage for the pus, and injection of carbolic acid lotion.

RUSH MEDICAL COLLEGE. May 23, Surgical Clinic. DR. GUNN. *Two Cases of Pott's Disease of the Spine—Mammary Abscess—Removal of Cystic Tumor of Right Shoulder.*

In regard to mammary abscesses, Dr. Gunn often advised a cold current of air, or the application of the ice bag, or an evaporating lotion, as an effective preventive of inflammation and suppuration. Often a tobacco ointment prevented the formation of milk in the breast.

In the removal of the cystic tumor, an incision of two inches was made in its long axis, and the cyst dissected. It was a simple cyst, the size of a hazel nut, and had been annoying the patient for eight years. Dr. Gunn used in the sutures white waxed silk, which is the ordinary saddler's silk, boiled in bleached white wax, heated over a water bath. The silk does not absorb fluids any more than wire, and is much easier to use than wire.

---

RUSH MEDICAL COLLEGE. Clinic for Nervous Diseases. Dr. WALTER HAY. *Three Cases of Progressive Muscular Atrophy.* Reported by HENRY L. HARRINGTON.

1st Patient. Man; aged 55; gardener. States that he has been much exposed to cold and wet. Knows of no case of the same disease in any of his ancestors. Has not had syphilis, neither has he been intemperate. Was well until six months ago. At that time noticed a dull aching pain in the left leg, also that the leg became weak. In a short time it began to decrease in size and continued getting smaller and weaker, until he was obliged to give up work. General health good; appetite good; bowels regular. Neither special sensation nor intellection impaired. On examination, find extensive atrophy of the extensor muscle, also of those of the calf. Patient is unable to flex the foot upon the leg, and the toes drag in walking. The anterior border of the tibia is sharply defined; there is also marked anæsthesia, and decrease in temperature of the leg and foot. He was ordered the following: R. Olei Morrhuæ, oz. ss.; Strychniæ Sulph., gr. 1-20, three times daily.

2nd Patient. Man; aged 48; laborer. States that for the last fifteen years he has worked in a packing house during the winter seasons, being engaged in cutting meat, using a cleaver weighing thirty pounds. In warm weather worked as a hod carrier; gives no history of syphilis, or of intemperance; was well until two years ago; at that time noticed pain, and also a creeping sensation beneath the skin, in the arms and shoulders. One year ago his arms began to get weak and to decrease in size; eyesight became weak and hearing defective; appetite was poor; bowels costive; urinated with difficulty, at times could not breathe easily; has continued getting worse ever since. On examination, find no constitutional disturbance; there is marked atrophy of the supra and infra spinati and deltoid muscles; the bony processes of the shoulder are very prominent. No paralysis; muscles of the forearm and hand not affected. Ordered—Strychnia Sulph., gr. 1-20, three times daily; good food. Also, to avoid violent exercise and fatigue.

3rd Patient. Man; aged 27; laborer. States that for several years he has worked in a warehouse, and has been obliged to do heavy lifting. Had syphilis in 1862. Has been intemperate for ten years. Was well until six months ago; at that time began to have pain in the back of the neck; also, a creeping and twitching sensation in the arms, which has persisted until the present time. Soon his arms and shoulders became weak, and the muscles began to decrease in size; suffered from headache; sight and hearing remained good and the intellect unimpaired; appetite good; bowels costive; urinates ten or twelve times daily; does not sleep well. On examination, find extensive atrophy of supra and infra spinati, deltoid and pectoral muscles; also, of those of the arm, forearm and hand, especially of the adductors of the thumb. [Presenting on both sides the "*main en griffe*" of Duchenne.] Patient cannot dress himself nor convey food to his mouth, although all power of motion is not lost. He was ordered Potassium Iodide, grs. x, with Fl'd Ext. Secale Cornut., dr. ss., three times daily; also, the use of electricity in the form of the continued current.

REMARKS.

Gentlemen: These patients illustrate exceedingly well this grave disorder. The disease is primarily due to granular degeneration and atrophy of trophic nerve cells: those cells which preside over nutrition. It is characterized by atrophy of single muscles, or of groups of muscles, generally commencing in the hand, sometimes in the shoulder. The first changes noticed in the microscopic appearance of the muscle, are that it becomes paler in color, and the transverse striæ partially disappear. As the disease progresses, the true muscular elements disappear entirely, leaving simply the sheaths intact, which become condensed into a tendinous cord. Its causes are probably, amongst others, syphilis, intemperance, excessive muscular labor, especially when combined with exposure to cold and wet.

Prognosis is exceedingly grave. Recovery rarely occurs. The disease spreads from muscle to muscle, finally involving those of respiration and deglutition, and the patient dies either of some intercurrent lung affection, of asphyxia, or of inanition. Its duration varies from two to ten years. Sometimes it is held in check for a time, but sooner or later recurs, and progresses to a fatal termination.

Little need be said in regard to treatment, as it is very unsatisfactory. The indications are, to increase nutrition, which is best done by increasing supply of blood to the parts; also to preserve the electrical contractility of the muscles. For this purpose the daily use of electricity is necessary, either the continued or the interrupted current, as the case requires. If any constitutional taint is present, it should be removed.

---

**Central Dispensary.** *Stammering, Loss of Voice and Neuralgia, from Constipation.* Service of Dr. NORMAN BRIDGE.

Master W. B., aged 15 years, of good growth and appearance, was brought to the Dispensary for treatment on March 24th. One week previously he had been suddenly noticed to stammer slightly. At the same time he complained of pain in his head and breast, especially when he would try to speak. The stammering, at first insignificant, grew rapidly worse, and in five days it became impossible for him to speak without great suffering. For two days he had used a slate and pencil with which he had made his communications, not speaking at all. Once, when his parents tried to compel him to talk, they say he had a fit. His general health was not impaired, yet his appetite was less than formerly, and as near as could be learned he had been somewhat constipated for some time. On asking him to reply to a question, he put his hand to his head, seemed in great agony, and half cried, while in a hitching, stammering way, and with great effort and labor, he answered with a monosyllable. What he said was said distinctly, and there was no misuse of language or aphasia. On quizzing his father it was learned that he did not always stammer. Once, in a fit of anger, he had spoken naturally, and once, also, on suddenly waking from sleep. There was no pain anywhere when at rest, no paralysis. The pupils, pulse and respiration were natural.

In commenting on the case, Dr. Bridge said there was no proof of the existence of any organic lesion anywhere. The fact that the stammering had been momentarily stopped by the emotions of anger and surprise, suggested strongly its functional character. In sensitive natures, it had long been known that such symptoms were producible by reflex irritation; by disturbance of distant organs, as by teething, constipation, worms or other local irritations. In this case there was no known source of reflex irritation except, possibly, the constipation. There might be worms, but we had no proof of it. He felt sure constipation alone might produce the phenomena of the case.

The boy was ordered powders of calomel and rhubarb (1 and 3 grs. respectively), one to be taken every three hours until thorough catharsis was produced, with instructions to return in two days.

The patient did not report for five days. He could then speak naturally and with ease, and he was well. All his symptoms had disappeared at once on the free movement of his bowels. In the meantime he had refreshed his memory, and said that at the time of his first visit his bowels had not moved for four or five days.

